# Nutritional and potential health benefits of chufa oil, olive oil, and anhydrous milk fat against gallstone disease in a C57BL/6N mouse model

**DOI:** 10.3389/fnut.2024.1445484

**Published:** 2024-09-26

**Authors:** Mohsen A. Zommara, Seham Swelam, Enrique Raya-Álvarez, Katsumi Imaizumi, Ahmed Elmahdy, Dalal A. Alkhudhayri, Abeer A. Aljehani, Ahmad Agil, Ehab Kotb Elmahallawy

**Affiliations:** ^1^Department of Dairy Science, Faculty of Agriculture, Kafrelsheikh University, Kafr El-Sheikh, Egypt; ^2^Department of Rheumatology, Hospital Universitario San Cecilio, Granada, Spain; ^3^Laboratory of Nutrition Chemistry, Division of Bioresource and Bioenvironmental Sciences, Graduate School, Kyushu University, Fukuoka, Japan; ^4^Department of Dairy Science, Faculty of Desert and Environmental Agriculture, Matrouh University, Matrouh, Egypt; ^5^Department of Home Economics, Prince Sattam Bin Abdul Aziz University, Al-Kharj, Saudi Arabia; ^6^Department of Food and Nutrition, Faculty of Human Sciences and Design, King Abdulaziz University, Jeddah, Saudi Arabia; ^7^Department of Pharmacology, School of Medicine, Biohealth Institute Granada (IBs Granada) and Neuroscience Institute, University of Granada, Granada, Spain; ^8^Grupo de Investigación en Sanidad Animal y Zoonosis (GISAZ), Departamento de Sanidad Animal, Facultad de Veterinaria, Universidad de Córdoba, Córdoba, Spain; ^9^Department of Zoonoses, Faculty of Veterinary Medicine, Sohag University, Sohag, Egypt

**Keywords:** C57BL/6N mice, lipid type, cholesterol, gallstone disease, aortic lesion

## Abstract

Dietary lipids play a major role in many diseases, particularly cardiovascular diseases. Recently, the health value of plant oils, particularly heart health, has been recognized. Despite these facts, limited information is available on the potential nutritional and anti-arteriolosclerosis effects of chufa oil, olive oil, and anhydrous milk fat in C57BL/6N mice. In the present study, the effects of olive oil (OO), chufa oil (CO), and anhydrous milk fat (AMF) on 4-week-old C57BL/6N male mice, a model for studies of diet-induced atherosclerosis, were investigated. The AIN-93G-based diet was supplemented with 15% of either OO, CO, or AMF. The final mixture of the diets contained 15% fat, approximately 1.25% cholesterol, and 0.5% sodium cholate. The data obtained showed that most mice had gallstone disease. The highest percentage of the gallstones formed were found in AMF groups (approximately 85.7% of the mice). However, the lowest one was found in the chufa oil group (42.9%), followed by the olive oil group (57.1%). Although the mice’s food intake significantly differed, their body weights did not change during the feeding period. The diet supplemented with CO resulted in a significant reduction in serum cholesterol compared with the other groups. Livers from the CO-fed group showed higher triglyceride levels than those from the AMF group. No significant differences were found in atherosclerotic lesions in the aortic valve between the groups. Collectively, our results show no deleterious nutritional effects of the fats used on C57BL/6N mice fed cholesterol-rich diets. Chufa oil improved cholesterol metabolism and atherogenic index in mice. However, the major issue is the formation of gallstones in all mice, which is most prominent in AMF, followed by olive oil and chufa oil diets.

## 1 Introduction

Chufa (*Cyperus esculentus*), also known as tiger nut, is a weed that grows in tropical and Mediterranean climates. Its tubers are commonly consumed in West and Central Africa in several ways, including raw, water-soaked, dried, and combined with roasted ground nuts (1). Ancient Egyptians were aware of the significance of the crop and cultivated it for both culinary and medicinal uses. Chufa is mostly used in Spain to produce the “Horchata de chufa,” a milk-like beverage that specializes in the Valencia region (2). Chufa tubers had an average chemical makeup of moisture 7.10%, protein 6.20–8.0%, fat 23.99–25.00% (70% oleic acid), ash 1.81%, total carbohydrates 60.90% (34% starch, 16% sucrose, and 10% fibers), and 7% other substances. Moreover, the amounts of the isoflavones daidzein and genistein in chufa tubers were 12.38 g/g and 8.46 g/g, respectively, and also contained about 1.68 mg/g oil of phytosterols (3, 4). Therefore, it is not surprising to mention that these tubers are used in traditional medicine in many regions of Africa and India, and even the dried tubers are ground and used to make bread and other bakery products (5, 6). Chufa oil has drawn the attention of many researchers and nutritionists because of its similarity, to a considerable extent, to olive oil in its chemical composition and nutritional value (7). Notably, the fatty acid composition and positional distribution of fatty acids in the triglycerides of oils are identical (8). However, the flavors and colors were not the same. Vitamin E levels are similar in both oils; however, chufa oil contains a higher concentration of plant sterols (3, 9). Chufa oil, like olive oil, has high concentrations of oleic acid (omega-9, 26.90%), linoleic acid (omega-6, 24.56%), and linolenic acid (omega-3, 0.27%), which are responsible for many of the well-known health advantages. This is one contributing factor to the increased interest in the health benefits of this oil (10). Research has indicated that oleic acid suppresses hunger and May help prevent obesity, atherosclerosis, and hypertension (10, 11). Studies have also demonstrated the anti-inflammatory properties of olive oil, potentially explaining its effectiveness in treating rheumatoid arthritis (12). Currently, the culinary sector primarily uses tiger nuts to create edible oils through cold pressing (4). It is commonly grown because it is easy to grow, and the extracted oil is sold as edible oil and fuel (13–15). In the cosmetic sector, tiger nut oil is highly valued and is a component of natural lotions, hand and body soaps, and lotion bars. It also hydrates and nourishes the skin, making it an excellent choice for epidermal treatments (16, 17).

Anhydrous milk fat (AMF) is a pure form of milk fat. In Egypt, it is made by boiling salted butter made from sour cream. This results in a clear fatty product with a distinctive flavor, physical structure, and texture after practically all moisture has evaporated and SNF has precipitated. AMF are characterized by a high percentage of saturated fatty acids (SFA), unlike chufa and olive oil, which are rich in monounsaturated fatty acids (MUFA, 63.26%). It contained 60% SFA, 24% MUFA, and 5% PUFA (18, 19). Milk fat contains a significant amount of short-chain fatty acids as well as trace amounts of branched fatty acids. High levels of saturated fatty acids are considered undesirable as they can increase the concentration of low-density lipoproteins (LDL), affect the ratio of LDL to high-density lipoproteins (HDL), and promote the proliferation of clothing and vascular smooth muscle. Increased linoleic and linolenic acid consumption through diet increases HDL cholesterol and lowers LDL cholesterol, whereas oleic acid decreases LDL cholesterol and does not affect HDL cholesterol levels (20). The relative types and amounts of dietary lipids consumed are thought to be extremely important because lipids play a crucial role in cardiovascular diseases, cancer, obesity, and diabetes (21, 22).

Atherosclerosis, a chronic inflammatory disease, is the main cause of most cardiovascular diseases. Studies have examined atherogenic processes in animals of all sizes, including both small and large animals. No model is perfect because each has its own advantages and limitations when it comes to changing the atherogenic process and emulating the human atherosclerosis or lipoprotein profiles. It is well known that different inbred mouse strains are likely to develop atherosclerosis when fed a special diet that encourages hyperlipidemia. Lipoprotein metabolism is one of the most distinct differences between mice and humans. Mice are used as high-density lipoprotein (HDL) models because they transport cholesterol mostly in HDL particles rather than in LDL, similar to humans. Mice have significantly lower cholesterol levels, which confer protection against atherosclerosis. On the other hand, Paigen (23) reported that the HDL level in the C57Bl/6 strain is considerably lower, which under distinct conditions makes it most prone to developing diet-induced atherosclerosis. In addition, when given an atherogenic diet, C57Bl/6 mice develop diabetes and obesity and are more sensitive to lesion development (24–26). Cholesterol gallstone disease is one of the most common conditions in the gastrointestinal tract and is caused by the complex interaction of multiple genetic and environmental factors that contribute to gallstone formation (27–29). Increased biliary production of cholesterol in the liver, which results in cholesterol-supersaturated bile, is the main contributor to gallstone development. Biliary cholesterol then crystallizes as cholesterol monohydrate microcrystals in the gallbladder, where it develops and aggregates to create macroscopic stones (30, 31).

There is a wealth of information available on the relationship between nutrition, gallbladder function, and cholesterol gallstone production. Several studies have highlighted the significance of plant-derived bioactive molecules in the creation of functional foods, which not only contribute to a healthy diet but also aid in the treatment of various metabolic disorders (31). However, little information is available on the potential effects of chufa oil, olive oil, or anhydrous milk fat on C57BL/6N mice fed an atherogenic diet. This study evaluated the effect of feeding C57BL/6N mice an atherogenic diet containing chufa oil, olive oil, or anhydrous milk fat. We examined the growth parameters of the mice, lipid profiles of their blood and liver, formation of gallstones, and occurrence of atherosclerosis in their aortic roots.

## 2 Materials and methods

### 2.1 Ethical statement

The experimental protocol of the present work was carried out under the control of the guidelines for animal experiments of the Faculty of Agriculture and Graduate Course at Kyushu University and Law no. 105 and notification no. 6 of the Government of Japan combined with the approval of the ethical committee of the Faculty of Agriculture, Kafrelsheikh University, Egypt, with approval number KFS-2021/8.

### 2.2 Materials

Olive oil was obtained from Nacalai Tesque, Inc. (Osaka, Japan). Unsalted cow sweet butter was purchased from a local market in Fukuoka, Japan. Chufa tubers were obtained from a local market in Tanta City, Egypt. It was milled to a fine powder using a laboratory electric mill for oil extraction.

### 2.3 Preparation of anhydrous milk fat and chufa oil

Chufa oil and AMF were prepared by vigorously shaking either butter or milled chufa with three volumes of hexane for an hour in a laboratory shaker. The mixture was centrifuged at 3,000 rpm for 5 min to separate the hexane layer. The extraction process was performed thrice with the solid residue. The hexane layers were then removed and combined. Fat was obtained by hexane evaporation under reduced pressure in a rotary evaporator and stored under argon gas at −30°C until use (32).

### 2.4 Diets and animals

The animals used in this study were 21 male, 7-weeks-old C57BL/6N mice with a mean body weight of 19–22 g obtained from Seac Yochitomi Ltd. (Yochitomi-Cho, Chikujyo-gun, Fukuoka-Ken-Japan). The mice were housed individually in plastic cages in a temperature-controlled room (22–25°C) with a 12 h-light/ 12 h-dark cycle. The animals were raised under the previous conditions for 4 weeks before starting the experiment. During this period, mice were fed a commercial non-purified diet (NMF, Oriental Yeast Co., Tokyo, Japan). Deionized water was provided *ad libitum*.

Animals were divided into three groups (seven mice each) in a randomized block according to their body weight and fed AIN-93G-based experimental diets (33) for 12 weeks. The diets were designed to contain equal amounts of energy (4,278 kcal/kg diet) (Table 1). As shown in Table 1, the final mixture of the diets contained 15% of olive oil (Nacalai Tesque, Kyoto, Japan), chufa oil or AMF, 1.25% cholesterol, and 0.5% sodium cholate. Therefore, the diets are considered atherogenic according to Wang et al. (34). Food and Deionized water were freely available throughout the experimental period. Body weight and food intake were recorded every alternate day.

**Table 1:** Composition of the AIN 93G experimental diets (g/kg).

Ingredients	OO	CO	AMF
Casein	200	200	200
Olive oil	150	–	–
Chufa oil	–	150	–
Anhydrous milk fat	–	-	150
Vitamin Mix	10	10	10
Mineral Mix	35	35	35
Choline bitartrate	2.5	2.5	2.5
L-cystine	3	3	3
Cellulose	50	50	50
α-corn starch	132	132	132
Corn starch	300	300	300
Sucrose	100	100	100
TBHQ	0.014	0.014	0.014
Cholesterol	12.5	12.5	12.5
Cholate-Na	5	5	5

OO, olive oil; CO, chufa oil; AMF, anhydrous milk fat; TBHQ, tertiary butylhydroquinone.

### 2.5 Tissue preparation

After food was withheld for 12 h (from 9:00 pm to 9:00 am), the mice were euthanized, and blood was withdrawn from the right ventricle under anesthesia with an intraperitoneal injection of sodium pentobarbital (5 mg/g body weight). Blood was collected and centrifuged at 3,000 rpm for 30 min for serum preparation. The liver, spleen, and brain were excised, washed in saline solution, weighed, and the relative weight % was calculated (g tissue/100 g body weight). The livers and serum were immediately immersed in liquid nitrogen and kept at −25°C until analysis. The hearts and aortas were dissected for histological examination.

### 2.6 Morphometric determination of atherosclerosis

To determine the cross-sectional lesion volume, hearts containing aortic roots were processed for a quantitative atherosclerosis assay, as previously described (35, 36).

### 2.7 Analysis methods

The fatty acid composition of dietary fat was determined using GLC as described by Imaizumi et al. (37). The concentration of vitamin E in chufa oil was determined using high-performance liquid chromatography (HPLC) (Waters 600E, Japan Millipore, Tokyo) according to the method described by Zommara et al. (38). The oil atherogenic index (AI) and thrombogenic index (TI) were used to evaluate the quality of fatty acids according to Ulbricht and Southgate (39). The method published by Testi et al. (40) was used to calculate the hypocholesterolemic to hypercholesterolemic (H/H) fatty acid ratio index, which takes into account the particular effects of fatty acids on cholesterol metabolism. The following equation was used to calculate the indices:

AI = [(C12:0 + (4 × C14,0) + C16:0)]/ (∑MUFA + ∑n-6 + ∑n-3).

TI = (C14:0 + C16:0 + C18:0)/ [(0.5 × ∑MUFA) + (0.5 × ∑n-6) + (3 × ∑n-3) + (∑n-3/∑n-6)].

H/H = (C18: 1c + C18: 2 n-6 + C20: 4 n-6 + C18: 3 n-3 + C20: 5 n-3 + C22:5 n-3 + C22:6 n-3)/ (C14:0 + C16:0).

where ∑MUFA = sum of monounsaturated fatty acids, ∑n-6 = sum of n-6 fatty acids, and ∑n-3 = sum of n-3 fatty acids.

Serum total cholesterol, triglycerides, high-density lipoprotein (HDL) cholesterol, and phospholipid concentrations were measured using commercially available enzyme assay kits (Cholesterol C-test, triglyceride G-test, and phospholipids B-test purchased from Wako Pure Chemical Industries Ltd. Osaka, Japan, and HDL-C2 test was from Daiichi Chemicals, Tokyo, Japan). Serum low-density lipoprotein (LDL) cholesterol concentration was calculated using the equation of DeLong et al. (41) as follows: LDL-cholesterol = Total cholesterol-HDL-cholesterol (0.16 × triglycerides). The atherogenic index plasma (AIP) was also calculated as described previously (42) using the following equation: AIP = log (triglycerides/HDL-cholesterol). The atherogenic index (AI) was calculated using the following equation (43): AI = [(Total cholesterol-HDL-cholesterol)/HDL cholesterol], Liver lipids were extracted as described by Folch et al. (44) and used to determine cholesterol (45), triglycerides (46), and phospholipids (47). Gallstone disease was confirmed by visual examination of mouse gallbladders with the naked eye.

### 2.8 Statistical analysis

SPSS (version 10.0) was used for statistical analysis. Each value is expressed as mean ± SE, of three replicates (48).

## 3 Results and discussion

### 3.1 Fatty acid composition of the experimental lipids

**Figure 1:**
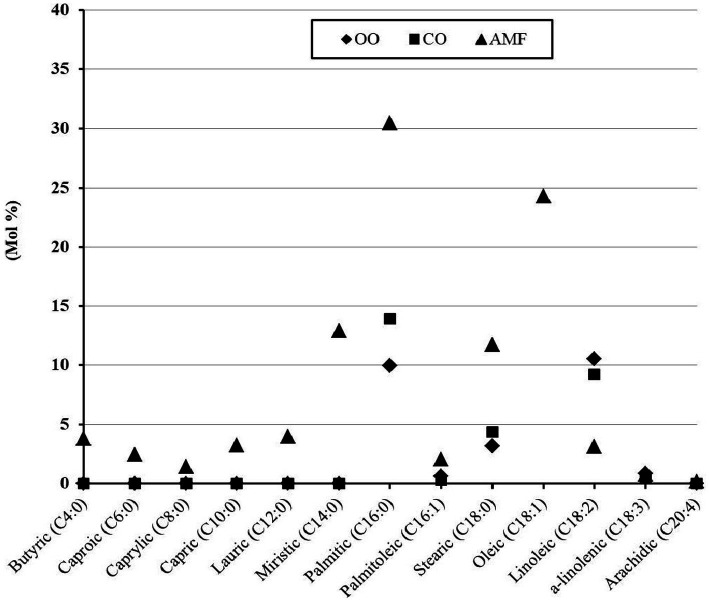
Fatty acid profile of olive oil (OO), chufa oil (CO) or anhydrous milk fat (AMF).

Figure 1 shows the fatty acid profile of the experimental lipids. It seems that olive and chufa oils had almost similar content of fatty acid profiles, with a predominant concentration of monounsaturated fatty acids (MUFA) (72.0–75.5%), most of which is oleic acid (71.8–74.8). Palmitic acid was significantly higher in chufa oil than in olive oil. Saturated fatty acids (SFA) comprised 13.2 and 18.3%, while polyunsaturated fatty acids (PUFA) accounted for 11.4 and 9.6% of olive oil and chufa oil, respectively (Table 2).

**Table 2:** Fatty acid composition (mol %) and vitamin E-content (mg/100 g) of the experimental lipids.

Fatty acid	OO	CO	AMF
SFA	13.18	18.28	69.74
MUFA	75.45	72.09	26.37
PUFA	11.37	9.63	3.89
USFA/SFA	6.59	4.47	3.21
AI	0.11	0.17	2.82
TI	0.29	0.44	3.17
H/H	8.65	5.85	0.66
Vitamin E	37.9	18.6	3.21

OO, olive oil; CO, chufa oil; AMF, anhydrous milk fat; AI: Oil atherogenic index; TI, thrombogenic index; H/H, hypocholesterolemic to hypercholesterolemic index.

Similar results were reported in a previous study (49–51) reported that chufa oil has the same fatty acid profile as olive oil, with oleic acid being the most common fatty acid in both oils. Oleic acid value content in olive oil ranges from 56 to 85%, but it varies from 65.5 to 76.1% in chufa oil (51–53). Palmitic acid, linoleic acid, and stearic acid are the other three significant fatty acids found in chufa oil (51, 54). In addition, the percentages of SFA, MUFA, and PUFA ranged between 13.2–16.6, 73.0–80.9, and 5.1–10.7, respectively, for olive oil, and 11.6–22.3, 65.6–76.1, and 9.2–13.6 for chufa oil, respectively (51–53, 55, 56).

On the other hand, the animal-derived anhydrous milk fat (AMF) contained significantly higher SFA (69.7%), lower MUFA (26.4%) and PUFA (3.89%) compared to plant-derived oils. The Previous studies reported that AMF contained 65.64–68.72 SFA, 27.40–29.46% MSFA, and 2.49–4.05 PSFA (21, 57, 58). The atherogenic index (AI) and thrombogenic index (TI) were considered as negative effects of the presence of C12:0, C14:0, and C16:0 acids in fats. Based on the AI and TI values, judgments about fat quality from the perspective of the human diet are possible. AI shows the correlation between the total amount of SFA and the total amount of UFA. Except for C18:0, the primary classes of SFA, C12:0, C14:0, and C16:0, are thought to be pro-atherogenic, and they seem to encourage the adherence of lipids to cells in the circulatory and immune systems (59, 60). In contrast, UFA has been reported to be anti-atherogenic because of its ability to prevent plaque buildup and lower levels of phospholipids, cholesterol, and esterified fatty acids (59, 60). Consequently, consuming meals or items with a lower AI can lower the levels of LDL and total cholesterol in human blood plasma a (61). The TI describes the thrombogenic potential of FA, which refers to the propensity for blood clots to form in blood vessels, and provides information on the contributions of various FA, which indicates the relationship between the pro-thrombogenic FA (C12:0, C14:0, and C16:0) and the anti-thrombogenic FAs (MUFA and the n-3 and n-6 families) (39). H/H characterizes the relationship between hypocholesterolemic fatty acids (cis-C18:1 and PUFA) and hypercholesterolemic fatty acids based on research on dietary FA and the control of plasma LDL (62). Comparable results of AI and TI were found for olive and chufa oils, and they had significantly lower values of these indices for AMF, which is advantageous from a health standpoint. Markiewicz-Keszycka et al. (57) reported that the values of AI and TI of cow’s milk fat were 2.55 and 3.22 g/100 g in order. In addition, a previous study (63) reported that the AI value of cow’s milk ranged from 1.88–4.18, while the TI value was 2.05–4.03 and H/H had a value of 0.032 to 0.74. It was also found that the vitamin E content of olive oil was twice that of chufa oil, as the first contained 37.9 mg/100 mL, while the other contained 18.6 mg/100 mL oil. AMF showed a significantly lower value (3.21 mg/100 mL) of vitamin E compared to olive and chufa oils. These results agree with those of previous studies (57, 64) that the content of vitamin E is significantly higher in olive oil than in chufa oil, reaching 260 mg/g in olive oil compared to 120.1 mg/g in chufa oil (54).

### 3.2 Gallstone disease

**Figure 2:**
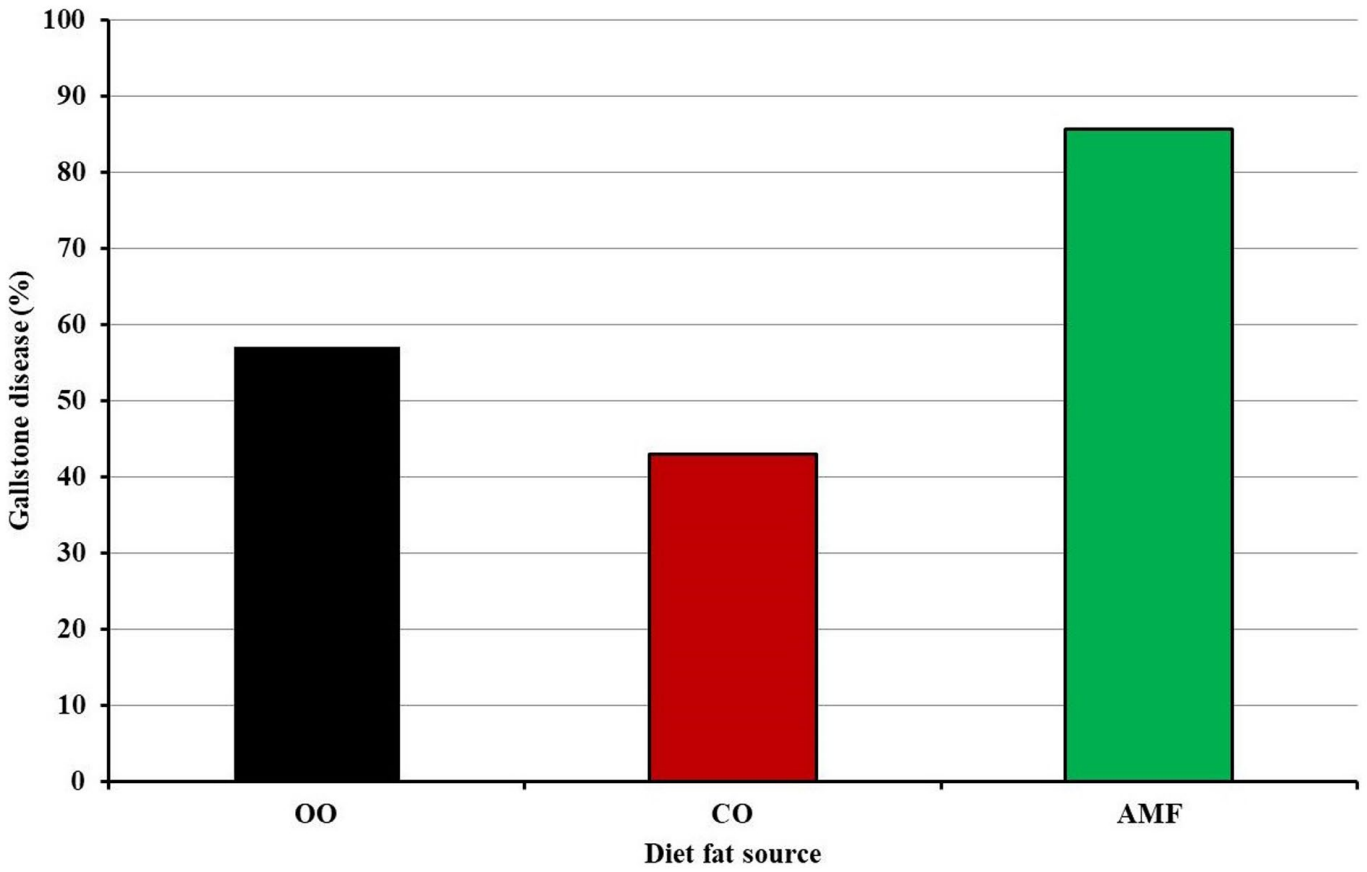
Percent of gallstone disease among mice fed olive oil (OO), chufa oil (CO) or anhydrous milk fat (AMF).

In the present study, C57BL/6N mice were fed a purified diet designed according to the AIN-76TM formula containing 15% either olive, chufa oil, or AMF and supplemented with 1.25 and 0.5% of cholesterol and cholic acid, respectively. Therefore, it was considered a lithogenic diet. Several studies have shown that C57BL/6 J mice develop gallstones when fed a lithogenic diet (65, 66). It can be detected (Figure 2) that about 62% of the mice (13 out of 21 mice) suffered from gallstone formation. The highest percentage (85.7%) of gallstone formation was found in AMF fed group (six out of seven mice), followed by olive oil with 57.1% (four out of seven mice). However, the lowest percentage was observed in the chufa group with a percentage of 42.9% (three out of seven mice). The reduction in gallstone formation in the mice fed the plant oils compared to that in mice fed the AMF diet May be attributed to the phytosterol content (67, 68) and fatty acid composition (69–71). The phytosterol profile of tiger nut oil is different from that of olive oil because it contains 168 mg/g of phytosterols compared to 100 mg/g in olive oil (8, 72).

Several animal studies have suggested that animals fed monounsaturated fatty acids (MUFA), mainly oleic acid (70) and polyunsaturated fatty acids (PUFA), May have a decreased risk of developing cholesterol gallstones than those fed saturated fatty acids (73, 74). Epidemiological investigations have demonstrated the preventive effect of MUFAs against gallstone disease (GD) in humans (70, 73, 74). In contrast, several studies have revealed that people with GD consume more total lipids rich in saturated fatty acids (69, 75, 76). In this respect, the decrease in gallstone formation in the mice fed olive and chufa oils compared to those fed AMF May be attributed, in part, to their fatty acid composition. Unlike AMF, the experimental oils were rich in MUFA 72–75% and PUFA (9.6–11.0%) with a low percentage of saturated fatty acids (SAF) (13–18%). Therefore, the susceptibility of the mice fed the AMF May be attributed to its high SAF (70%) and low MUFA (26%) and PUFA content on one side, in addition to the containing plant sterols on the other side. A previous study (77) stated that pure butter contained 99.71% cholesterol of total sterols, whereas the amount of *β*-sitosterol, a marker for phytosterols, was zero.

### 3.3 Mice growth parameters

**Figure 3:**
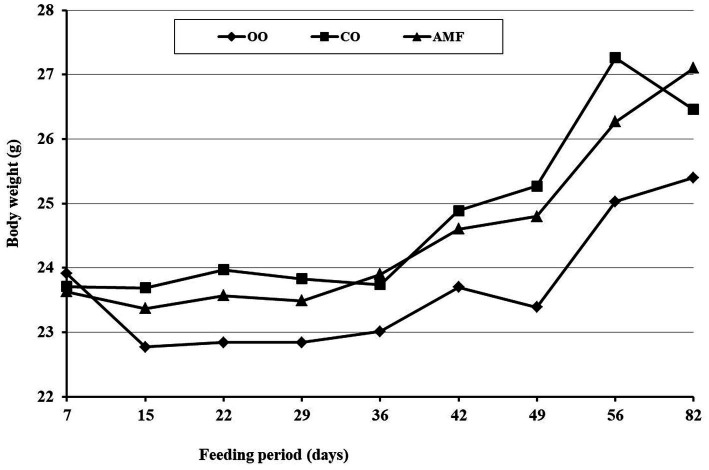
Body weight development of mice fed olive oil, chufa oil or anhydrous milk fat during the feeding period. Data are means of 7 mice per group.

There was no significant effect of the type of dietary fat on the growth characteristics of the mice (Table 3), which May be attributed to the energy equilibrium (4,278 kcal/kg diet) among all diets. The daily food intake of the mice was significantly higher in chufa oil (CO) and AMF groups than in (OO) fed, which affected their body weight development during the feeding period (Figure 3). As shown in Figure 3, all mice showed slight body weight development during 12 weeks of feeding. As shown in Table 3, unlike the oils, feeding on AMF led to a significant increase in mouse body weight gain, although the food efficiency (g gain/g food) was comparable among all groups. The AMF-fed mice had a significantly higher relative spleen weight (%) than the other groups. However, the liver and brain weights (%) showed no significant differences among the mice (Table 3). These results reflect the deleterious effect of the lithogenic diet on mouse growth, which suppresses the development of mouse growth. In this respect, our previous study (78) demonstrated an increase of 9 and 12 g in the weight gain of C57BL/6N mice fed on a non-atherogenic diet containing 15% of either olive oil or pure milk fat (Ghee) for 5 weeks, respectively. It was expected that the mice’s growth rate would be low during the feeding time, as the mice were adults at the beginning of the purified diet feeding (5 weeks old). Additionally, a diet high in cholesterol and cholic acid May produce hepatocyte toxicity in mice, leading to a negative impact on their growth (79).

**Table 3:** Growth parameters of mice fed olive oil, chufa oil or anhydrous milk fat.

Parameters	OO	CO	AMF
Initial body weight (g)	23.9 ± 0.46 ^a^	23.7 ± 0.45 ^a^	23.6 ± 0.52 ^a^
Final body weight (g)	25.4 ± 0.67 ^a^	26.5 ± 0.80 ^a^	27.1 ± 0.46 ^a^
Body weight gain (g)	1.49 ± 1.08 ^a^	2.74 ± 0.90 ^a^	3.47 ± 0.70 ^a^
Food intake (g/day)	2.91 ± 0.07^b^	3.27 ± 0.06^a^	3.28 ± 0.12^a^
Food efficiency	0.006 ± 0.004 ^a^	0.010 ± 0.003 ^a^	0.013 ± 0.002 ^a^
Spleen (%)	0.34 ± 0.02^b^	0.37 ± 0.03^ab^	0.47 ± 0.06^a^
Liver (%)	6.85 ± 0.26 ^a^	7.36 ± 0.33 ^a^	6.65 ± 0.42 ^a^
Brain (%)	1.76 ± 0.05 ^a^	1.71 ± 0.04 ^a^	1.67 ± 0.03 ^a^

OO, olive oil; CO, chufa oil; AMF, anhydrous milk fat; food efficiency, g gain/g food. Data are presented as the mean ± SE for 7 mice per group. ^a,b^ Means with different superscripts within a row are significantly different at p ≤ 0.05.

### 3.4 Serum lipid profile

Table 4 shows serum lipid profile of mice fed OO, CO, or AMF. Feeding mice on CO diet significantly reduced serum total cholesterol (174.2 mg/dL) compared to OO (242.3 mg/dL), however, the AMF oscillates between them (231.3 mg/dL). The same trend was found for serum LDL-cholesterol concentration; whereas CO resulted in 134.7 mg/dL compared to 202.9, and 180.1 mg/dL in OO and AMF-fed mice, respectively. On the other hand, the AMF-fed mice resulted in a significant increase in serum HDL-cholesterol (45.6 mg/dL) compared to OO (33.8 mg/dL) and CO (34.4 mg/dL) fed groups. The type of fat does not affect serum triglycerides which were comparable among all mice (32.5–35.2 mg/dL). No significant differences were found in serum phospholipid content between OO (168.3 mg/dL) and CO (151.5 mg/dL) mice groups however, the AMF-fed mice resulted in significantly higher phospholipid content (208.2 mg/dL). The calculated AIP and AI of mice serum assumes a protective effect of CO and AMF against atherogenicity compared to OO. According to a previous meta-analysis of randomized controlled trials examining the effects of olive oil on blood lipid levels, compared to other plant oils, the obtained data showed that, increasing the consumption of olive oil can lower serum TC, LDL-cholesterol, and TAG and increase HDL-cholesterol (80). On the other hand, a diet rich in tiger nut oil May improve the concentration of good (HDL) cholesterol and reduce the risk of cardiovascular-related illnesses (73). Also, consumption of milk fat can have various effects on the plasma lipid profile. Milk fat is rich in saturated fatty acids, which are known to increase LDL cholesterol levels when consumed in high amounts. High LDL cholesterol is a risk factor for cardiovascular disease. However, milk fat also contains other components such as monounsaturated and polyunsaturated fatty acids which have been associated with lower levels of LDL cholesterol and higher levels of HDL cholesterol. These fats also contain beneficial nutrients like vitamin D and calcium that May contribute to overall heart health. Overall, the effect of milk fat on the plasma lipid profile May vary depending on individual differences in metabolism, overall diet, and lifestyle factors. It is important to consume dairy products in moderation as part of a balanced diet to maintain a healthy plasma lipid profile (81–83).

**Table 4:** Serum lipid profile of mice fed olive oil, chufa oil or anhydrous milk fat.

Parameters	OO	CO	AMF
Total cholesterol (mg/dl)	242.3 ± 28.3^a^	174.2 ± 16.7^b^	231.3 ± 17.0^ab^
HDL-cholesterol (mg/dl)	33.8 ± 2.30^b^	34.4 ± 2.63^b^	45.6 ± 3.26^a^
LDL-cholesterol (mg/dl)	202.9 ± 28.2^a^	134.7 ± 14.8^b^	180.1 ± 16.1^ab^
Triglycerides (mg/dl)	34.9 ± 2.90 ^a^	32.5 ± 3.39 ^a^	35.2 ± 2.14 ^a^
Atherogenic index plasma (AIP)	0.01 ± 0.04 ^a^	−0.03 ± 0.05 ^a^	−0.11 ± 0.03 ^a^
Atherogenic index (AI)	6.4 ± 1.0^a^	4.1 ± 0.3^b^	4.1 ± 0.4^b^
Phospholipids (mg/dl)	168.3 ± 11.2^ab^	151.5 ± 7.67^b^	208.2 ± 19.7^a^

OO, olive oil; CO, chufa oil; AMF, anhydrous milk fat. Data are presented as the mean ± SE for 7 mice per group. ^a,b^ Means with different superscripts within a row are significantly different at *p* ≤ 0.05.

It has been widely documented that the atherogenic index of plasma (AIP) is a reliable predictor and biomarker of cardiovascular disease (CVD). The AIP has been used to evaluate blood lipids and as an effective CVD and dyslipidemia indicator using log (TG/HDL-C) (84, 85). According to Dobiasova (42) AIP levels between −0.3 and −0.1 indicate low, 0.1–0.24 indicates medium, while values over 0.24 indicate high cardiovascular (CV) risk. Consequently, compared to mice fed chufa oil (−0.03) or AMF (−0.11), the mice fed olive oil (0.01) had higher AIP values, which May indicate a higher risk of developing coronary heart disease (CHD). The same trend was observed when AI was calculated according to Kawase et al. (43). There was a significant reduction in AI by feeding CO (4.1) and AMF (4.1) to mice compared to that in the OO group (6.4). Therefore, these results suggest a protective effect of CO and AMF against atherosclerosis.

### 3.5 Liver lipid profile

**Figure 4:**
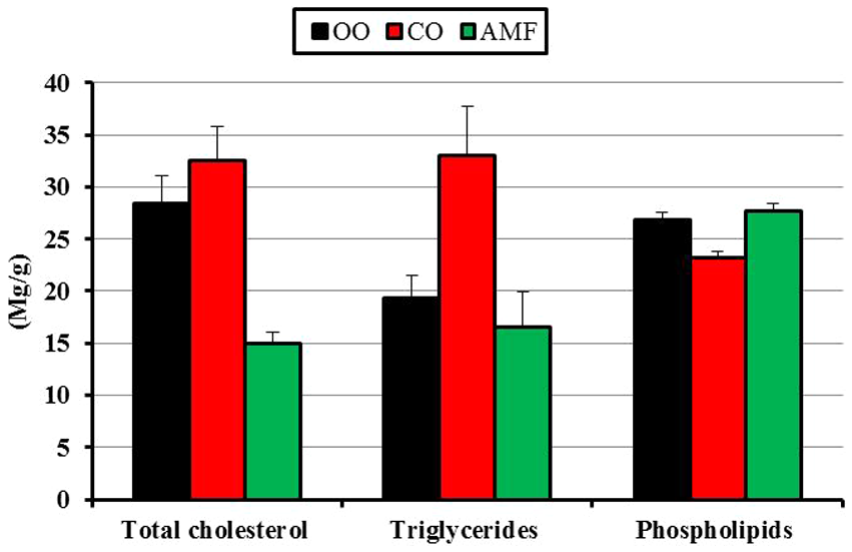
Liver lipid profile of mice fed olive oil (OO), chufa oil (CO) or anhydrous milk fat (AMF). Data are presented as mean ± SE for 7 mice/ group. ^a,b^ Means with unlike superscripts are significantly different at *p* ≤ 0.05.

The liver lipid profiles of mice are shown in Figure 4. Livers from the AMF-fed mice resulted in significantly lower cholesterol content (14.9 mg/g) compared to those fed on OO (28.4 mg/g) or CO (32.5 mg/g). Kumar (81) fed Wistar rats a nutritionally balanced AIN-76 diet for 8 weeks with varying amounts of ghee (0.25–10%). They found that when ghee was added to the diet at concentrations higher than 2.5%, the serum lipid profiles of these animals demonstrated a dose-dependent decrease in triglyceride levels, total cholesterol, and low-and very-low-density lipoprotein cholesterol. Desmarchelier et al. (86) stated that C57BL/6N mice fed a Western diet containing 34% fat for 12 weeks displayed elevated plasma cholesterol levels due to the higher dietary cholesterol intake, whereas liver and intestinal cholesterol levels were noticeably lower. They proposed that to adapt to high dietary fat consumption, the liver and intestines activate *de novo* cholesterol production and other cholesterol-saving systems, as well as undergo significant alterations in phospholipid metabolism. No significant differences were observed in triglyceride concentrations between the OO (19.3 mg/g) and AMF (16.5 mg/g) groups. However, a significant increase was observed in the CO diet (33.0 mg/g). In contrast, the content of phospholipids in the liver decreased significantly in the case of the CO diet (23.2 mg/g), whereas OO and AMF recorded higher values (26.8 and 27.7, respectively) with insignificant differences. According to previous studies, cirrhotic individuals exhibit aberrant lipid metabolism, particularly low cholesterol levels and hypobetalipoproteinaemia (87). A previous study (88) revealed that people with liver cirrhosis exhibit reduced lipid levels, including total cholesterol, triglycerides, LDL, and HDL.

### 3.6 Aortic valve lesions

**Figure 5:**
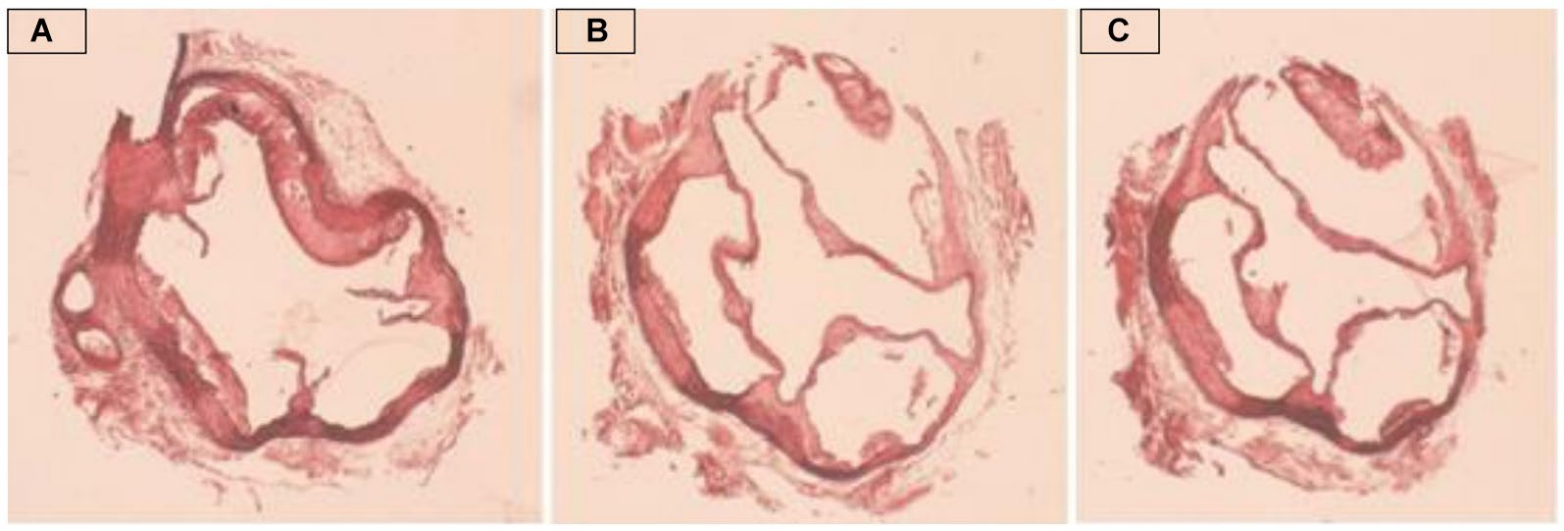
Representative photomicrographs of hematoxylin and eosin (H&E)-stained cross-sections of the aortic root of C57BL/6N mice fed OO **(A)**, CO **(B)** or AMF **(C)**.

**Figure 6:**
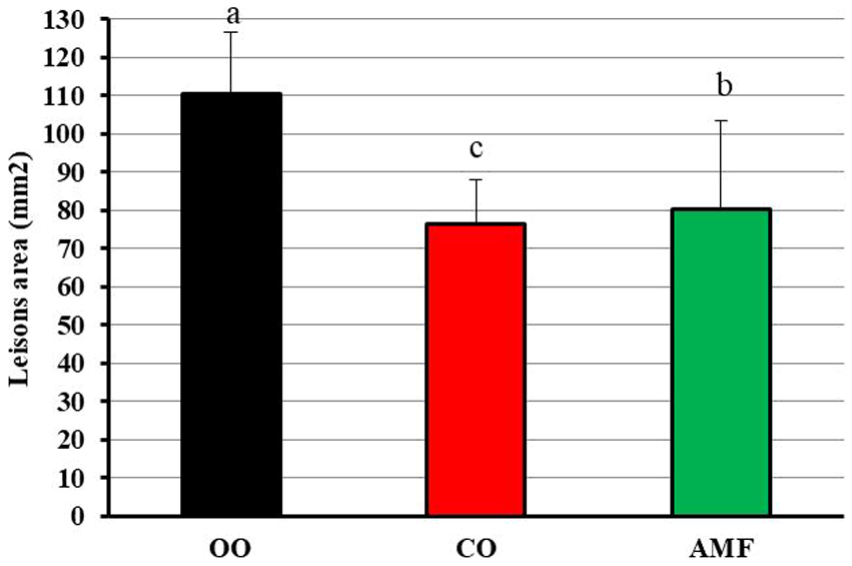
Atherosclerotic lesions (mm^2^) in the aortic arch of C57BL/6N mice fed on olive oil (OO), chufa oil (CO) or anhydrous milk fat (AMF). Data are presented as mean ± SE. ^a,b^ Means with unlike superscripts are significantly different at *p* ≤ 0.05.

A light microscope was used to visually inspect aortic valve lesions. Figure 5 shows representative photomicrographs of hematoxylin and eosin (H&E)-stained cross-sections of the aortic roots of C57BL/6N mice fed OO (A), CO (B), or AMF (C). The age of C57pl/6 N mice is a key factor in the development of atherosclerosis (Simo). The mice raised on atherogenic diets for 12 weeks were 23 weeks old at the beginning of the feeding period. Therefore, we examined the mice for atherosclerosis when they were approximately 6 months old. As shown in Figure 5, all mice suffered from atherosclerotic lesions to varying extents depending on the dietary fat type (Figure 6). The rats fed olive oil tended to have the highest degree of sclerosis lesions, whereas those fed chufa oil had the lowest. Extensive literature exists on the differing susceptibilities of inbred mouse strains to atherosclerosis during feeding a modified diet that promotes hyperlipidemia. A previous study (36) reported that C57BL/6 mice were the most susceptible to the development of diet-induced atherosclerosis among the examined inbred strains. The obtained results could be linked to the calculated AIP and AI data in Table 4, which indicate a high risk for the OO mice group to develop atherosclerosis compared to CO or AMF mice. In this study, Paigen et al. (89) demonstrated that C57BL/6 J female mice developed aortic lesions at each intercostal artery, at the aorta-heart junction, and in sporadic regions encompassing 1.1% +/− 0.5 (SD) of the aortic surface after 14 weeks on an atherogenic diet including 1.25% cholesterol, 15% fat, and 0.5% cholic acid. After 9 months of the atherogenic diet, the mice developed large lesions near the heart and intercostal arteries, affected 8% +/− 3 (SD) of the remaining aorta, and discovered lesions in the coronary arteries. On the other hand, another study (23) found no conclusive link between the formation of gallstones and atherosclerosis.

## 4 Conclusion

Feeding C57 Pl/6 N mice with plant or milk fats in an atherogenic diet (high in cholesterol) for 3 months had no significant effect on mouse growth. In addition to the importance of olive oil as one of the main features of the Mediterranean diet, which is recommended as an alternative to the Western diet rich in animal fats, the present study demonstrated the superior hypocholesterolemia and anti-atherogenic effects of chufa oil compared to olive oil and milk fat. All mice developed gallstones; however, plant oils showed a protective effect, which May be attributed to their phytosterol content. In addition, all mice developed atherosclerotic lesions, which were more evident in mice fed with milk fat. Therefore, these findings May prove the possibility of using chufa oil to produce healthy functional foods as an alternative to olive oil.

## Data Availability

The original contributions presented in the study are included in the article, further inquiries can be directed to the corresponding author.
